# Contributing to Overall Life Satisfaction: Personality Traits Versus Life Satisfaction Variables Revisited—Is Replication Impossible?

**DOI:** 10.3390/bs8010001

**Published:** 2017-12-23

**Authors:** Bernd Lachmann, Rayna Sariyska, Christopher Kannen, Konrad Błaszkiewicz, Boris Trendafilov, Ionut Andone, Mark Eibes, Alexander Markowetz, Mei Li, Keith M. Kendrick, Christian Montag

**Affiliations:** 1 Molecular Psychology, Institute of Psychology and Education, Ulm University, Helmholtzstrasse 8/1, D-89081 Ulm, Germany; rayna.sariyska@uni-ulm.de (R.S.); christian.montag@uni-ulm.de (C.M.); 2 Department of Computer Sciences, University of Bonn, 53012 Bonn, Germany; info@ckannen.com (C.K.); konrad.blaszkiewicz@gmail.com (K.B.); trendafilov.boris@gmail.com (B.T.); ionut.andone@gmail.com (I.A.); mark.eibes@gmail.com (M.E.); alex@iai.uni-bonn.de (A.M.); 3 Counseling Centre, Beijing University of Civil Engineering and Architecture, Beijing 100044, China; amorelm415@gmail.com; 4 Clinical Hospital of Chengdu Brain Science Institute, MOE Key Laboratory for Neuroinformation, University of Electronic Science and Technology of China, Chengdu 610051, China; k.kendrick.uestc@gmail.com

**Keywords:** well-being, model of life satisfaction, integrative approach, cross-cultural, replication crisis, personality

## Abstract

Virtually everybody would agree that life satisfaction is of immense importance in everyday life. Thus, it is not surprising that a considerable amount of research using many different methodological approaches has investigated what the best predictors of life satisfaction are. In the present study, we have focused on several key potential influences on life satisfaction including bottom-up and top-down models, cross-cultural effects, and demographic variables. In four independent (large scale) surveys with sample sizes ranging from *N* = 488 to 40,297, we examined the associations between life satisfaction and various related variables. Our findings demonstrate that prediction of overall life satisfaction works best when including information about specific life satisfaction variables. From this perspective, satisfaction with leisure showed the highest impact on overall life satisfaction in our European samples. Personality was also robustly associated with life satisfaction, but only when life satisfaction variables were not included in the regression model. These findings could be replicated in all four independent samples, but it was also demonstrated that the relevance of life satisfaction variables changed under the influence of cross-cultural effects.

## 1. Introduction

During the last few decades, life satisfaction has been investigated by an impressive number of studies around the world. Research to date has focused on several different areas: firstly, the association between personality and (overall) life satisfaction has been investigated, although findings have been inconsistent [1,2,3,4,5,6,7,8]; secondly, researchers have also explored the link between overall life satisfaction and various life satisfaction variables such as health, job, income, housing, leisure, and family [9,10,11,12,13]. The associations between various life satisfaction variables and overall life satisfaction are complex [9] as well as inconsistent. Other studies have investigated potential associations between physical variables and life satisfaction such as fitness activities [14,15,16] or one’s own personal health situation [17,18,19]. Moreover, the presence of a disease [20,21,22] and its impact on life satisfaction have been explored. Generally, fitness activities and good health have a positive influence on life satisfaction, whereas individuals suffering from a disease usually show lower levels of life satisfaction. Furthermore, several studies have investigated cross-cultural differences in the context of life satisfaction [23,24,25] along with the influence of demographic variables such as age and gender [26,27]. These latter studies have provided some (preliminary) evidence that cross-cultural differences as well as age and gender could have an influence on life satisfaction, but more research is needed to establish clear patterns. This brief overview of studies concerning life satisfaction highlights both the worldwide interest in this research topic as well as the need for further research to determine the key influential factors. 

In the literature, the terms life satisfaction, happiness, and subjective well-being are often used interchangeably. Although this might not be entirely correct, it is understandable given that these terms overlap to a certain degree [28]. The construct of happiness plays a key role worldwide as impressively evidenced by ongoing projects such as “The World Happiness Report” (http://worldhappiness.report/). This project demonstrates that happiness is pursued by most individuals [28]. Although being happy seems to be of immense importance for most humans, this construct is scientifically still difficult to frame [29]. While happiness is the overarching term used, well-being could be interpreted as a more distinct part of it [30] and hence can be better framed and analyzed. According to Diener et al. [31] subjective well-being (SWB) can be further subdivided into affective and cognitive components. Here positive and negative affect are related to emotional aspects of subjective well-being, whereas life satisfaction represents the cognitive part of subjective well-being [32]. This is how we will refer to life satisfaction in the present study. Understanding variables impacting upon life satisfaction could clearly lead to a more profound understanding of the broader terms of well-being and happiness [33].

Typically, life satisfaction is measured with the help of short questionnaires such as the Satisfaction with Life Scale (SWLS) [31] or so-called “single items measures” as used in the German Socio-Economic Panel (SOEP) [34]. An example of such a single item measure would be “How satisfied are you with your life overall?” or, in the case of specific life satisfaction variables, “How satisfied are you with your health?” The results of both types of measurement (short questionnaires and single item measures) have been shown to be very similar [35]. In the present research, we used single-item measures as used in the SOEP. Therefore, our study design also allows us to take a closer look at the relationship between specific life satisfaction variables and overall life satisfaction. 

In addition to the research described above, two approaches/theories in life satisfaction research have been discussed intensely in recent years: bottom-up versus top-down theories [11,30,33,36]. Bottom-up theories consider overall life satisfaction as a function of various areas of life satisfaction [33]. From this perspective, many different areas with a potential influence on overall life satisfaction [11] can be identified in the literature, such as satisfaction with health, job, income, housing, leisure time, and family [11,13]. In contrast, researchers have also proposed a top-down approach where life satisfaction is determined by personality disposition (which manifests in rather stable cognitive and emotional traits resulting in an individual displaying stable behavior—see Montag & Panksepp, 2017 for an overview).

The bottom-up perspective of life satisfaction implies that overall life satisfaction is a complex function of various life satisfaction variables which are usually not simply additive [9]. When individuals are asked about their overall life satisfaction the outcome on this measure depends, amongst others, on factors such as individuals giving greater importance to a certain area of life satisfaction compared to others, or also on personal life preferences [33]. For example, a person with a high attachment motivation will most probably appraise satisfaction with family and work differently compared to an individual with a preference for achievement (this example clearly also hints at the top-down approach, since personality traits are closely linked to individual differences in motivational aspects). The complex relationship between areas of life satisfaction and overall life satisfaction can be further explained with mechanisms termed compensation, spillover, and segmentation effects [9]. A compensation effect implies a negative, whereas a spillover effect assumes a positive association between areas of life satisfaction variables and/or overall life satisfaction [33]. In the first case, a change in one area, for example one’s job, would lead to an inverse change in another area, for example one’s family (being more satisfied with the job could reflect less time for family issues). In the latter case, a change in one area would cause an equal change of life satisfaction in another area (e.g., being healthy also results in higher overall life satisfaction). The term segmentation indicates that changes in one area have no effect on other areas and/or overall life satisfaction [33]. Further detailed information concerning the nature of the relations between the areas of life satisfaction and overall life satisfaction can be found in Rojas et al. [9].

The top-down perspective considers the level of overall life satisfaction or areas of life satisfaction as a function of personality and other stable traits [11,30]. In this context, dispositional factors would determine the extent to which a person feels satisfied, allowing an inference on the degree of satisfaction by analyzing their personality structure. For example, the investigation of extraversion or neuroticism could give researchers an idea how satisfied a person should be: Here, mainly positive associations between extraversion and life satisfaction have been observed, whereas the link between neuroticism and life satisfaction is usually negative [30]. These kinds of relations have also been confirmed by several meta-analyses [2,37]. On the other hand, factors beyond personality traits are also considered of importance. For example, situational factors such as (critical) life events or other environmental influences have been shown to be of relevance to assessing the level of life satisfaction. A recent meta-analysis demonstrated an effect of life events especially on cognitive well-being [38]. Taken together, these findings suggest that neither a bottom-up nor top-down perspective alone can sufficiently explain well-being. Instead, an integrated view combining both perspectives might be most successful. Although both approaches are often described as competing theories [11], there are also several proposals to integrate both the bottom-up and top-down perspectives in one integrated model [13,37,39,40]. Erdogan et al. (2012) stated that top-down effects (e.g., personality) could influence the perception of different areas of life satisfaction, and in doing so also affect overall life satisfaction. He also suggested considering personality as a distal predictor in models of life satisfaction and not as a control variable. Nevertheless, there are variables that should be controlled for in models of life satisfaction such as age, gender, and education [30,41]. Several findings indicate that these variables could modify the association between personality and/or specific life satisfaction variables with overall life satisfaction [26,27,30,42], although findings are not always consistent.

Cross-cultural differences between collectivistic and individualistic cultures, concerning life satisfaction, have also been observed [23,32,43]. Notably, it can be assumed that life satisfaction is interpreted differently in different cultures: In an individualistic culture, individuals are rather focused on their own goals, interests, and feelings and not so much on the well-being of a group (such as friends or family). In contrast, in more collectivistic cultures, harmonious relationships with other people are of higher value than personal goals. This could, for example, result in a higher appraisal for family satisfaction in collectivistic compared to individualistic cultures [24]. In addition, differences concerning the level of life satisfaction across nations have also been found: People in individualistic cultures general report higher levels of life satisfaction than ones in collectivistic cultures. One explanation for this finding is that the personal, goal-oriented view in individualistic cultures contributes to more self-referred attribution of failure and success, possibly leading to greater overall life satisfaction compared to individuals in collectivistic cultures [11].

The aim of the present study was to (re-)investigate if and how strong factors, such as personality, different areas of life satisfaction, demographic variables, and cross-cultural effects, are associated with overall life satisfaction to help contribute towards a basic working model of life satisfaction. In line with this, a combination of both bottom-up and top-down approaches was chosen for the first survey. The potential influence of age and gender on overall life satisfaction was also examined (Survey 1). This first survey is of particular interest, since we used a smartphone application to assess life satisfaction and personality variables. As such, if our results were found to be similar to the existing literature, future researchers might be encouraged to use this approach more often in life satisfaction research to assess life satisfaction on a large-scale level. To replicate and confirm our results in Survey 1, we conducted a second survey with a very similar setup and the same research question (Survey 2). To further rule out any doubt concerning the validity of the very short questionnaire used to measure personality (Big Five Inventory 10 (BFI-10); please refer to the method section for further details), we repeated the analyses with a third sample this time using the NEO-FFI [44] to gather information on personality (Survey 3). This approach helped to demonstrate that personality associations with life satisfaction are not influenced by the chosen questionnaire. Finally, to be able to explore cross-cultural differences in life satisfaction, a last survey was conducted in China (the first three studies were conducted in Europe). For this final survey, we once more also assessed the psychometric quality of BFI-10 by administering/comparing both the BFI-10 and NEO-FFI in the context of life satisfaction (Survey 4).

## 2. Materials, Methods, and Results

In the next section we will report the methods and results for all of our four surveys separately before we analyze all results together in a combined discussion section.

### 2.1. Materials and Methods for Survey 1

The aim of the first survey was to investigate the association between life satisfaction and personality. We also investigated the influence of various life satisfaction variables on overall life satisfaction. 

To achieve a large sample, we used a specifically developed smartphone application called “Menthal” [45]. “Menthal” was designed to track smartphone usage, giving its user a quantitative feedback on his/her behavior. Furthermore, it was possible for the subscribers to fill in several short online questionnaires targeting areas of life satisfaction and personality that we could then analyze (see Section 2.1.2).

#### 2.1.1. Participants

A total of *N* = 40,297 participants (*N* = 14,874 females) filled in the questionnaires and provided socio-demographic information such as age, gender, and education. The mean age for the whole sample was *M* = 24.45 (*SD* = 9.99) ranging from 10 to 90 years. A total of 3.6% had no school leaving certificate, 11.1% had a secondary school leaving certificate, 57.1% had a Baccalaureate-Diploma, and 28.2% had a university degree. Since “Menthal” was developed for smartphones running Android 4.0 (Google, Mountain View, CA, USA) (Ice Cream Sandwich) and higher, but not for any other operating system, participation in this survey was restricted to Android users (of note, recent research has demonstrated that users of platforms such as Android and iOS (Apple Inc. Cupertino, CA, USA) do not differ much in their personality [46]). The application could be downloaded free via Google Play store (Google, Mountain View, CA, USA) (https://play.google.com/store/apps/details?id=open.menthal). Participants did not receive monetary compensation, but they did receive a detailed personal feedback on their smartphone behavior as an incentive. The present survey was completely anonymous and approved by the local ethics committee of the University of Bonn, Bonn, Germany. Every participant had to give their electronic consent prior to their participation in the survey and when downloading the application.

#### 2.1.2. Materials

Data on personality were collected by administering a very short questionnaire called Big Five Inventory 10 (BFI-10) [47] with a Likert scale ranging from 1 (“disagree strongly”) to 5 (“agree strongly”). The development of this questionnaire was mainly based on the Big Five Inventory 44 (BFI-44) [48], which consists of 44 short items. The BFI-10 is composed of only ten of these items with two items covering each of the personality dimensions. To ensure the operational capability of the BFI-10, its creators [47] focused on the comparability between BFI-10 and BFI-44. Overall, they found substantial correlations, ranging from *r* = 0.74 (agreeableness) to *r* = 0.89 (extraversion), as well as an acceptable test–retest stability for the BFI-10 of *r* = 0.75 (BFI-44: *r* = 0.83). The authors of the study [47] noted that the BFI-10 with less than a quarter of the items of the BFI-44 still had sufficient psychometric power to adequately measure personality in research settings.

Life satisfaction was measured via items retrieved from the German Socio-Economic Panel (SOEP). One section of the panel covers the current life situation and assesses several different areas contributing putatively to overall life satisfaction. For this survey, we asked for the degree of satisfaction in the following areas and order: health, job, income, housing, leisure time, and overall satisfaction with life. Following a recommendation of the SOEP, the question for overall satisfaction with life was asked at the end of the life satisfaction questionnaire to avoid possible interference with specific life satisfaction variables [34]. It is important to mention that overall life satisfaction is not a simple composite of various life satisfaction variables. In fact, all life satisfaction items are distinct, but also overlap to some extent (e.g., a person more satisfied with his leisure might also have as consequence a higher score on overall satisfaction). The questionnaire items were all answered, using a Likert scale, ranging from 0 (“completely dissatisfied”) to 10 (“completely satisfied”).

To retrieve the data, we used the above-mentioned smartphone application “Menthal”. The application was developed at the University of Bonn by computer scientists of the Department of Computer Science (see co-authors on this paper). The app is able to track a multiplicity of variables concerning individual user behavior such as number of calls, duration of calls, specific application usage, and many more (please note that we also published a paper on WhatsApp usage and personality using data from some of the individuals included in the present sample [49] but the present study only considers their questionnaire data). With these data, “Menthal” provides users with detailed feedback about their smartphone usage behavior, which should work to motivate them to fill-in the questionnaires. Consequently, in return for using our free service, participants agreed to provide our research team with recorded variables on their smartphone usage (not relevant for the present survey), personality data, information on their life satisfaction, as well as socio-demographic data. Every user received feedback on their questionnaire data only after filling in items on personality and so on. In this way, we were confident that users should be motivated to give true answers.

#### 2.1.3. Procedure

After an extensive testing phase, “Menthal” was made available at the Google Play Store. To attract broad attention for our survey, a press release was made concerning the capabilities of our smartphone application. Subsequently, many radio and TV stations in Germany did cover this topic, attracting a high number of possible users. At the end of the first week after we had made “Menthal” available on the Google Play Store more than 10,000 participants had downloaded the application. Even though it was predictable that the excitement of the first phase would decrease, we nevertheless ultimately managed to include more than 40,000 users in this survey. The data collected from each user were transferred online to the servers of the Department of Computer Science (University of Bonn) where it was stored and processed. Finally, the raw questionnaire data were converted into SPSS files and used for the analyses.

#### 2.1.4. Statistical Analyses

SPSS version 22.0 for Windows (IBM SPSS Statistics, IBM, New York, NY, USA) was used to carry out the statistical analyses. All analyses were performed with the complete sample and a modified sample excluding participants younger than 18 years to control for a possible influence of age in the findings. The presence of a normal distribution for all variables was inspected visually as well as using the procedure described by Miles et al., 2001 [50]. Gender differences with respect to life satisfaction were investigated using *t*-tests. The associations between overall life satisfaction and life satisfaction variables, as well as those between personality and life satisfaction, were calculated using Pearson correlation analysis. A stepwise multiple regression model with overall life satisfaction as the independent variable was conducted to analyze the influence of age/gender, personality, and life satisfaction variables on overall life satisfaction. The stepwise method was used because of its exploratory character, since the literature is discordant on how life satisfaction is influenced (e.g., bottom-up versus top-down approach). Additionally, a hierarchical regression analysis was conducted to investigate in more detail the specific influence of demographic, personality, or life satisfaction variables on overall life satisfaction. The rationale for the block design is presented in the results section.

#### 2.1.5. Results for Survey 1

For all variables mean, standard deviation, minimum, maximum, and skew were calculated (Table 1). All variables were normally distributed [50], and we did not find any outliers. With respect to the variable “overall satisfaction with life,” a significant gender difference was observed (*t*_(40295)_ = 12.47, *p* < 0.001; *Cohens d* = 0.13) where females showed lower scores (*M* = 7.03; *SD* = 2.11) than males (*M* = 7.29; *SD* = 1.94). Furthermore, age and “overall satisfaction with life” were negatively correlated (*r_S_* = −0.10, *p* < 0.001). Consequently, gender (dummy-coded) and age were included into the stepwise multiple regression model. As a third demographic variable, we considered the education level of the participants (dummy-coded) in our regression. Conducting the analyses with the complete sample in contrast to the modified sample (participants younger than 18 years excluded) provided small value changes but no change in any of our findings. Therefore, we conducted the analyses and reported the results for the complete sample only. An overview about the distribution of all life satisfaction variables in the sample relating to age (total sample and separated by gender) can be found in Table S1.

#### 2.1.6. Correlations

Extraversion (*r* = 0.16, *p* < 0.001) and conscientiousness (*r* = 0.12, *p* < 0.001) were significantly correlated with the overall satisfaction score. A significant negative correlation was observed between neuroticism and overall satisfaction (*r* = −0.20, *p* < 0.001). The various life satisfaction variables were significantly correlated with the overall satisfaction score. The lowest correlation was found between satisfaction with the job/income and overall life satisfaction (*r* = 0.39, *p* < 0.001), and the highest one was between leisure and overall life satisfaction (*r* = 0.55, *p* < 0.001). The correlations observed between personality and specific satisfaction variables were rather weak. Extraversion and conscientiousness correlated positively with all specific satisfaction variables (ranging from *r* = 0.06 to *r* = 0.20, *p* < 0.001). Neuroticism showed a negative association with every single specific satisfaction variable (ranging from *r* = −0.11 to *r* = −0.23, *p* < 0.001). For openness to experience and agreeableness, the highest correlation was *r* = 0.08, *p* < 0.001. All correlations can be found in Table 2.

#### 2.1.7. Regression Model

Multi-collinearity was not a problem (lowest tolerance: 0.67; highest *VIF* = 1.47) and the data met the assumption of independent errors (Durbin-Watson value = 1.99) as well as the assumption of non-zero variances (variances between 0.65 and 99.89). Personality variables and specific satisfaction scores, which showed a significant correlation with the overall satisfaction score, as well as gender and age, were entered in the stepwise regression model. The independent variables explained 46% (*R*^2^ = 0.460) of the overall satisfaction score (*F*_(7,29410)_ = 3577.40, *p* < 0.001). The variable with the highest impact on the overall satisfaction score was leisure (beta = 0.36; *T* = 74.69, *p* < 0.001), followed by income (beta = 0.19; *T* = 36.95, *p* < 0.001). Furthermore, the variables health (beta = 0.16; *T* = 33.33, *p* < 0.001), housing (beta = 0.15; *T* = 31.68, *p* < 0.001), and job (beta = 0.08; *T* = 15.99, *p* < 0.001) contributed to the overall satisfaction score. Of note, demographic or personality variables did predict only a very minimal part of the overall satisfaction score. The combined change of *R*^2^ for these variables was only 0.04% in contrast to 45.7% for the specific satisfaction variables. Taking different theoretical approaches into account (bottom-up, top-down) the influence of demographic variables, personality, and life satisfaction variables on overall life satisfaction were also analyzed using a hierarchical regression model. Age, gender (dummy-coded), and education (dummy-coded) were entered in the first block, followed by personality variables in the second block (top-down) and life satisfaction variables in a third block (bottom-up). The values for *R*^2^ changed slightly with 0.7% for age and gender, and 6.8% for personality variables, but were still 38.7% for the life satisfaction variables. The results of the stepwise regression can be found in Table 3.

### 2.2. Material and Methods for Survey 2

The purpose of the second survey was to replicate the results found in the first survey. Again, we investigated the association between life satisfaction and personality, as well as the influence of various life satisfaction variables on overall life satisfaction using the same questionnaires. This time data were collected via electronic tablets instead of smartphones. The electronic tablets were available within the scope of a public exhibition, thereby giving many individuals the chance to participate in our survey.

#### 2.2.1. Participants

Overall *N* = 5000 participants (*N* = 2395 females) answered the questionnaires and provided socio-demographic information. The mean age of the total sample was *M* = 30.33 (*SD* = 19.86) ranging from 6 to 95 years. A total of 31.8% had no school leaving certificate, 30.8% had a secondary school leaving certificate, 14.9% held a Baccalaureate-Diploma, and 22.5% had a university degree. Since we did not use a smartphone application to collect the data (as in Survey 1), only participants who came to the exhibition could participate in the survey. Participation was completely anonymous and approved by the local ethics committee of the University of Bonn, Bonn, Germany. Every participant had to give their electronic consent prior to their participation in the survey. As an incentive, every participant received a personalized feedback on the basis of the provided information, but no monetary compensation.

#### 2.2.2. Materials

As in Survey 1, data on personality were collected using the BFI-10 [47]. The information on life satisfaction was gathered using the same items in the same order derived from the SOEP [34], as described in Survey 1.

Participants filled in the questionnaires via electronic tablets with custom-built software installed. This software guided the participants through the questions and later enabled individual feedback. The software used electronic sliders, scroll-bars, and buttons to allow a convenient completion of all forms.

#### 2.2.3. Procedure

We collected the data during an exhibition installed on a large boat that traveled through Germany and Austria. The theme of the exhibition was “digital society.” Several cities were selected as a place to host the exhibition allowing interested individuals to come and see the scientific exhibits, with no entrance fee charged. At each stop, the boat stayed for a few days before traveling to the next city. This approach was tailored to attract many individuals. At our stand, participants could fill-in various questionnaires including personality and life satisfaction using an electronic tablet. Participants received individual feedback, dependent on the responses they provided, as an incentive to make their responses as accurate as possible. For some participants (*N* = 3084), we also collected responses relating to individual usage of watch-based smartphones and associated overuse of digital technologies. The results of this survey—namely on “Zeitgeber” usage and PIU—are also published [51]. Furthermore, several participants also (*N* = 4852) filled in questionnaires concerning their individual Internet usage. This allowed us to investigate the association between life satisfaction and problematic Internet use [52]. All participants provided electronic consent for participation in the present study.

#### 2.2.4. Statistical Analyses

The same statistical analyses were carried out as in Survey 1.

#### 2.2.5. Results for Survey 2

During inspection of the data, we found an anomaly concerning the age variable. A total of *N* = 150 participants chose 0 or 99 as their age, which represents the lowest and highest available values on the age scale. Because age could be inserted using a slider, we assumed that those participants did fill in an incorrect age by either not moving the slider at all or simply moving it all the way across to the other side of the scale. At this point, we had doubts if the relevant participants provided reliable information at all. Since there was no possibility to investigate this issue further, we decided to exclude these participants (age 0 or 99) from any further analysis (3.00% of all participants). Moreover, two participants with a reported age of 1 and one participant with an age of 2 were excluded because it seemed unlikely that these participants did fill in the questionnaires by themselves. Additionally, participants recording ages of 6 (2 participants), 7 (6 participants), 8 (20 participants), and 9 (35 participants) were excluded from the sample due to concerns referring to the younger participants ability to understand (reading and writing abilities) and to correctly assess life satisfaction and personality items. Finally, from all datasets collected (*N* = 5000) *N* = 4784 datasets were used in the analysis because of our concerns regarding the age variable. Thereafter, the characteristics of the sample changed as follows: For age, the mean changed from *M* = 30.33 (*SD* = 19.86) to *M* = 29.27 (*SD* = 16.93) and the distribution of gender was altered from 52.1% (men) to 51.5% (men). Mean, standard deviation, minimum, maximum, and skew was calculated for all variables (Table 4). As in Survey 1, conducting the analyses with the complete sample in contrast to the modified sample (participants younger than 18 years excluded) provided small value changes but no change in any of our findings. Therefore, we conducted the analyses and reported the results for the complete sample only. All variables were normally distributed. For the variable “overall satisfaction with life,” a significant gender difference was observed (*t*_(4782)_ = 2.44, *p* = 0.015; Cohen’s *d* = 0.07)) where females showed lower scores (*M* = 7.66; *SD* = 2.14) than males (*M* = 7.81; *SD* = 2.13). Furthermore, age and “overall satisfaction with life” were negatively correlated (*r* = −0.09, *p* < 0.001). Gender (dummy-coded), education variables (dummy-coded), and age were included into the stepwise multiple regression model.

#### 2.2.6. Correlations

The highest positive correlation between personality variables and overall life satisfaction was observed between extraversion and overall life satisfaction (*r* = 0.16, *p* < 0.001). Between neuroticism and overall life satisfaction, the correlation was negative (*r* = −0.19, *p* < 0.001). The life satisfaction variables correlated to a higher extent than personality variables with the overall life satisfaction score (between *r* = 0.46, *p* < 0.001 for income and *r* = 0.61, *p* < 0.001 for leisure). As in Survey 1, the correlations between BFI-10 and life satisfaction variables were weak, apart from extraversion and leisure (*r* = 0.17, *p* < 0.001), as well as neuroticism and health (*r* = −0.21, *p* < 0.001). The complete list of correlations can be found in Table 5.

#### 2.2.7. Regression Model

Multi-collinearity was not a problem (lowest tolerance: 0.63; highest *VIF* = 1.51) and the data met the assumption of independent errors (Durbin-Watson value = 1.96) as well as the assumption of non-zero variances (variances between 0.25 and 288.37). As in Survey 1, personality variables and specific satisfaction scores with significant correlations to the overall satisfaction score as well as gender and age were entered in the stepwise regression model. The predictor variables explained 53% (*R*^2^ = 0.533) of the overall satisfaction score (*F*_(6,4383)_ = 834.95, *p* < 0.001). Again, leisure had the highest impact on overall life satisfaction (beta = 0.32; *T* = 25.69, *p* < 0.001), followed by housing (beta = 0.26; *T* = 20.29, *p* < 0.001). Additionally, the variables health (beta = 0.15; *T* = 11.99, *p* < 0.001), income (beta = 0.14; *T* = 12.03, *p* < 0.001), and job (beta = 0.12; *T* = 9.79, *p* < 0.001) contributed to the overall satisfaction score. As in Survey 1, neither demographic nor personality variables could predict a significant part of the overall satisfaction score. The combined change of *R*^2^ for these variables was 0.02% in contrast to 53.3% for the specific satisfaction variables. As before, we conducted a hierarchical regression analysis entering age and gender in a first block, followed by personality variables in a second block and life satisfaction variables in a third block. The values for *R*^2^ changed slightly with 0.9% for age and gender, 6.2% for personality variables, and 46.6% for life satisfaction variables. The results of the stepwise regression analysis can be found in Table 6.

### 2.3. Material and Methods for Survey 3

The results of our regression analyses in Surveys 1 and 2 showed an unexpectedly low influence of personality on life satisfaction. This is interesting, since some previous studies [5,53] reported fairly robust associations between life satisfaction and personality, and this has been additionally supported in a meta-analysis by DeNeve (*r* = 0.19) [2]. The findings of DeNeve are based on the analyses of 197 independent samples in which the relationship between a total of 137 different personality traits and life satisfaction have been investigated. Therefore, in Survey 3, we decided to investigate the same research question using another inventory, in order to rule out the possibility that our different results may have been due to the particular questionnaire used. We replaced the BFI-10 with the NEO-FFI [44], a reliable and well-established tool in personality research. Data were collected via an electronic database of the Department of Molecular Psychology/Institute of Psychology and Education at Ulm University, Ulm, Germany. After invitation by e-mail, participants filled in the questionnaires at home using their personal computers.

#### 2.3.1. Participants

A total of *N* = 496 participants (364 females) filled in the online questionnaires and gave socio-demographic information including age, gender, and education. The mean age in this sample was *M* = 23.08 (*SD* = 4.13), ranging from 18 to 55 years. A total of 4.4% had a secondary school leaving certificate, 69.0% held a Baccalaureate-Diploma, and 26.6% had a university degree. The recruiting was done at Ulm University in Germany. The participants were mainly students as well as their relatives and friends. Data on life satisfaction and personality in Survey 3 were collected within the scope of a much larger study. The expenditure of time to participate in the complete study was around three to four hours, and every participant received monetary compensation of 30 euro. The survey was approved by the local ethics committee of the Ulm University and every participant had to give their electronic and written consent to participate.

#### 2.3.2. Materials

In contrast to Surveys 1 and 2, here we used the NEO FFI [44] to assess personality. The NEO-FFI is a well-proven tool that measures the five dimensions of personality: neuroticism, extraversion, openness to experience, conscientiousness, and agreeableness. Cronbach’s alphas in our sample ranged from *α* = 0.76 (openness) to *α* = 0.85 (neuroticism). The NEO-FFI consists of 60 Likert scaled items ranging from 1 (“strongly disagree”) to 5 (“strongly agree”). In Survey 3, a German translation of the NEO-FFI [54] was used. Data on life satisfaction were gathered using the above-mentioned items extracted from the SOEP [34] together with one additional item: family. This item was inserted just before the one referring to overall life satisfaction. The main purpose of this still ongoing project is the collection of personal data via the online database together with gene samples for specific molecular analyses. However, for Survey 3, no molecular analysis was conducted.

#### 2.3.3. Procedure

Participants received a personalized link to the online questionnaire via email. Upon receipt of a personal link, participants could fill-in the questionnaires comfortably at home. After completion, the data were transmitted SSL encrypted to our secured data server and processed for further analyses.

#### 2.3.4. Statistical Analyses

The statistical analyses conducted in Survey 3 were similar to those in Surveys 1 and 2.

#### 2.3.5. Results for Survey 3

An inspection of the data did not reveal any missing information or other peculiarities such as outliers. Mean, standard deviation, minimum, maximum, and skew were calculated for all variables (Table 7). All variables were normally distributed other than age. No significant gender difference concerning overall life satisfaction was observed (*t*_(496)_ = −0.81, *p* = 0.42). Age, education variables, and overall life satisfaction did not correlate. Therefore, gender, education, and age were not included in the stepwise multiple regression model.

#### 2.3.6. Correlations

In line with the first two surveys, extraversion (*r* = 0.23, *p* < 0.001) showed the highest positive correlation with the overall satisfaction score. This was also the highest positive correlation between personality variables and overall life satisfaction. Neuroticism was again negatively correlated (*r* = −0.23, *p* < 0.001) with overall life satisfaction. All life satisfaction variables showed correlations with overall life satisfaction and leisure had the highest correlation (*r* = 0.56, *p* < 0.001). Between NEO-FFI variables and life satisfaction variables, weak to no correlations were observed, with the highest negative correlation being between leisure and neuroticism (*r* = −0.28, *p* < 0.001) and the highest positive correlation between extraversion and leisure *r* = 0.28, *p* < 0.001). For a detailed list of all correlations please refer to Table 8.

#### 2.3.7. Regression Model

Multi-collinearity was not a problem (lowest tolerance: 0.73; highest *VIF* = 1.37) and the data met the assumption of independent errors (Durbin–Watson value = 2.07) as well as the assumption of non-zero variances (variances between 0.27 and 15.26). The personality variables and specific satisfaction scores with significant correlations with the overall satisfaction score were entered in the stepwise regression model. The predictor variables explained 50% (*R*^2^ = 0.494) of the overall satisfaction score (*F*_(5,490)_ = 95.82, *p* < 0.001). Satisfaction with leisure explained the largest part of overall life satisfaction (beta = 0.29 *T* = 7.58, *p* < 0.001), followed by family (beta = 0.28 *T* = 7.31, *p* < 0.001). The variables health (beta = 0.22; *T* = 6.33, *p* < 0.001), housing (beta = 0.17; *T* = 4.89, *p* < 0.001), and income (beta = 0.07; *T* = 2.09, *p* = 0.037) also contributed to the overall satisfaction score. Since no personality variable was of relevance in the final regression model, the explanation of variance for the overall satisfaction score was derived only from the life satisfaction variables. Finally, a hierarchical regression analysis was conducted with personality variables in a first block and life satisfaction variables in a second block. For the personality variables *R*^2^ moved up to 7.8%. Nevertheless, *R*^2^ for life satisfaction variables was still much higher at 47.1%. The results of the stepwise regression can be found in Table 9.

### 2.4. Material and Methods for Survey 4

For the fourth survey, we collected data on life satisfaction and personality in China. The purpose of this survey was twofold: First, we were interested in the investigation of cross-cultural differences between collectivistic (China) and individualistic (Germany) cultures. Second, both the BFI-10 and the NEO-FFI were administered. Thus, here we could compare the results of both inventories simultaneously. The data acquisition for Survey 4 took place in Beijing, China.

#### 2.4.1. Participants

Four hundred eighty-eight participants (*N* = 192 females) filled in the online questionnaires and gave us socio-demographic information including age, gender, and education. The mean age of the whole sample was *M* = 19.60 (*SD* = 1.79) ranging from 18 to 26 years. A total of 0.6% had no school leaving certificate, 3.1% had a secondary school leaving certificate, 96.1% held a Baccalaureate-Diploma, and 0.2% had a university degree. The recruiting was done at the Student Counseling Centre, Beijing University of Civil Engineering and Architecture, Beijing, China. The participants were psychology students, students from other fields and relatives of the students and their friends. There was no financial or other compensation for the participants. The participation on this survey was anonymous and had ethical approval by the local ethics committee of the University of Peking, Peking, China. To participate, everyone had to give electronic and written consent.

#### 2.4.2. Materials

To gather information on personality, we used the BFI-10 and the NEO-FFI. Life satisfaction was measured using the same items as in Survey 3 (retrieved from the German Socio-Economic Panel; SOEP). The life satisfaction variables had to be modified slightly since almost all participants were students with no job. For that reason, we asked them to evaluate their courses instead of a non-existent job, arguing that studying at a university could be like having a job (variable study). Additionally, the question for income was altered to the amount of money subjects had available to them (variable money). Other than these two substitutions, the order of all items was the same as in Survey 3. The items of the SOEP and the BFI-10 (both were English versions) were translated into Mandarin Chinese by a native Chinese speaker with an excellent knowledge of the English language. Validation of the translation was accomplished via back-translation into the English language by a different person. We also administered a Chinese version of the NEO-FFI that was already translated and checked for its reliability in other studies [55,56] following the same procedure as outlined above. Cronbach’s alphas in our sample extended from *α* = 0.71 (openness) to *α* = 0.87 (neuroticism).

#### 2.4.3. Procedure

All participants for the survey were invited to visit the test facility at Beijing University of Civil Engineering and Architecture, Beijing, China. The recruitment was done on the campus via bulletin-boards and personal announcements in various lectures. At the test facility, all participants used digital devices to answer the questionnaires and gave written consent before participation.

#### 2.4.4. Statistical Analyses

Again, we conducted the same statistical analyses as outlined in Surveys 1–3.

#### 2.4.5. Results for Survey 4

We did not have any missing data or outliers. Mean, standard deviation, minimum, maximum, and skew were calculated for all variables (Table 10). All variables were normally distributed. No significant gender difference concerning overall life satisfaction was observed (*t*_(488)_ = −0.30, *p* = 0.766). Age, but not education, showed a weak correlation with overall life satisfaction (*r* = 0.09, *p* = 0.040). Therefore, only age was included in the stepwise multiple regression model.

#### 2.4.6. Correlations

For the NEO-FFI the highest positive correlations were observed between extraversion/conscientiousness (both *r* = 0.19, *p* < 0.001) and overall life satisfaction. A negative correlation was found between neuroticism and overall life satisfaction (*r* = −0.23, *p* < 0.001). The NEO-FFI variables did correlate with life satisfaction variables, with the highest negative correlation between neuroticism and study (*r* = −0.39, *p* < 0.001). Furthermore, neuroticism was negatively correlated with all other life satisfaction variables in contrast to openness, which correlated with none of the life satisfaction variables. The results for the BFI-10 variables were as follows: Between extraversion and overall life satisfaction no correlation was observed. The correlation between neuroticism and overall life satisfaction was negative (*r* = −0.11, *p* = 0.015). Conscientiousness (*r* = 0.10, *p* = 0.005), openness (*r* = 0.12, *p* = 0.009), and agreeableness (*r* = 0.16, *p* < 0.001) were positively correlated with overall life satisfaction. The correlations between BFI-10 and life satisfaction variables were rather weak except for neuroticism, which correlated negatively with every single life satisfaction variable (highest correlation with study *r* = −0.24, *p* < 0.001). All life satisfaction variables were significantly correlated with the overall satisfaction score. The lowest correlation was found between satisfaction with health and overall life satisfaction (*r* = 0.31 *p* < 0.001) and the highest correlation between family and overall life satisfaction (*r* = 0.55, *p* < 0.001). To get insights into the overlap between personality dimensions assessed with BFI-10 and NEO-FFI please refer to Table S2. A summary of all correlations including and life satisfaction can be found in Table 11 and Table 12, respectively.

The Chinese version of the BFI-10 showed an unexpected result (we observed no correlation between extraversion and overall life satisfaction which had previously been the case). A closer investigation of the items used to measure extraversion (Chinese version of the BFI-10) yielded a weak non-significant correlation between the item “… is reserved” and the total score for extraversion (*r* = 0.11, *p* = 0.056) and a weak significant correlation between the item “ ... is outgoing, sociable” and the total score for extraversion of the NEO-FFI (*r* = 0.11, *p* = 0.016). To give researchers the possibility to check our translation, we posted the Chinese version of the BFI-10 in Appendix A Of note, the back-translation of the Chinese word for “reserved” ended up in “undemonstrative”.

#### 2.4.7. Regression Model

Neither with NEO-FFI nor with BFI-10 variables was multi-collinearity a problem (lowest tolerance: 0.71 (0.72 with BFI-10); highest *VIF* = 1.40) and the data met the assumption of independent errors (Durbin–Watson value = 1.76 (1.79 with BFI-10) as well as the assumption of non-zero variances (variances between 0.21 (2.51 with BFI-10) and 6.38). In Survey 4, we additionally compared both personality questionnaires to assess the robustness of results from study 1–3 using only the BFI-10. We conducted a stepwise regression analysis for both the BFI-10 and the NEO-FFI. A complete summary referring to both questionnaires is displayed in Table 12. Similar to the other surveys, personality variables and specific satisfaction scores together with age were entered into the stepwise regression model. The predictor variables including NEO-FFI explained 45% (*R*^2^ = 0.447) of the overall satisfaction score (*F*_(6,481)_ = 64.82, *p* < 0.001). The explained variance of overall life satisfaction for the stepwise regression model with BFI-10 variables was 44% (*R*^2^ = 0.442; *F*_(6,481)_ = 63.44, *p* < 0.001). As far as the model including the NEO-FFI was concerned, family had the highest impact on overall life satisfaction (beta = 0.36; *T* = 9.15, *p* < 0.001) followed by money (beta = 0.22; *T* = 5.82, *p* < 0.001). Furthermore, the variables leisure (beta = 0.18; *T* = 4.75, *p* < 0.001), study (beta = 0.13; *T* = 3.51, *p* < 0.001), openness (beta = 0.10; *T* = 3.01, *p* = 0.003), and age (beta = −0.08; *T* = −2.34, *p* < 0.020) contributed to the overall satisfaction score.

The results for the stepwise regression model including the BFI-10 were very similar with the exception that age and openness switched positions in the final model. As before, neither demographic nor personality variables could predict a large part of the overall satisfaction score. The combined change of *R*^2^ using the NEO-FFI as a measure of personality and demographic variables was 1.8% (1.3% BFI-10) in contrast to 42.9% (42.2% BFI-10) for life satisfaction variables. In line with the other three surveys, a hierarchical regression analysis was conducted, entering age in the first block followed by personality variables in the second block and life satisfaction variables in a third block. The values for *R*^2^ changed slightly with 9.8% (5.4% BFI-10) for age and personality variables and 35.5% (39.2% BFI-10) for life satisfaction variables. An overview of the stepwise regression models can be found in Table 13.

## 3. Discussion

The aim of the present study was to extend research on the association between life satisfaction and several factors with proposed influence on life satisfaction and by combining two different approaches (bottom-up and top-down). To achieve this goal and find robust results, we used four different samples, which allowed us to first answer questions concerning the influence of the bottom-up and top-down approaches on life satisfaction [11,30,33,36] and gave us the chance to replicate our findings in multiple samples. The replication of the same findings in our different samples supports their robustness and provides proof against the replication dilemma in psychological research. Additionally, we obtained further insights concerning the quality of the BFI-10 and the presence of cross-cultural effects [23,32,43] concerning life satisfaction. 

A main finding of the present study was that the association between overall life satisfaction and personality variables [4,5,8,57] was not as strong as we expected. A stepwise multiple regression model considering demographic variables, personality variables, and life satisfaction variables as predictors of overall life satisfaction showed that only 0.1–1.8% of the variance of overall life satisfaction could be explained by demographic and personality variables. This finding could be replicated in all four surveys. When personality variables and life satisfaction variables were entered in a hierarchical regression model in separate blocks, the explained variance (*R*^2^) of all personality variables did increase to a maximum of *R*^2^ = 0.098, but this was still much lower than the highest *R*^2^ (0.533) of all life satisfaction variables. Since all variables were at least weakly correlated, different values in R^2^ when using a stepwise versus a hierarchical regression model can be explained with the shared variance between those variables. From this perspective, life satisfaction variables (bottom-up) have a much higher impact on the overall life satisfaction score compared to all the other predictor variables. However, this does not mean that the association of personality variables and overall life satisfaction should be ignored, since there were still moderate and significant correlations between personality and overall life satisfaction in our samples. This view is also supported by the meta-analysis of deNeve et al. [2]. For our findings, concerning the rather weak association between personality variables and overall life satisfaction we would like to suggest the following explanation. Diener et al. [58] has emphasized the necessity of investigating not only the direct link between personality and overall life satisfaction, but also interactional and indirect effects (such as the presence or absence of various life circumstances). Additionally, some authors have reported moderating or mediating effects of specific variables influencing the link between personality and overall life satisfaction. For example, Gutierez et al. [41] pointed out the need for considering demographic variables, and Magee et al. [26] discussed the influence of cultural background on personality and life satisfaction. Thus, the association between personality and overall life satisfaction seems to be a complex network with both direct and indirect pathways. Additionally, several further factors might have the potential to influence the association between personality and overall life satisfaction. These include, for example, the individual’s personal life circumstances, health situation, level of physical fitness, and the presence of diseases. If all these factors have a share in the determination of overall life satisfaction, a single factor would probably contribute only to a low extent to the overall life satisfaction score. Hence, the higher the number of predictors to determine a criterion, the more difficult it should be to find robust results. This is possibly one reason why the association between personality and overall life satisfaction was rather weak in the present study. In addition to this, we would like to emphasize that the substantial number of participants collected in Survey 1 could be achieved via a large-scale smartphone study. The fact that the same findings were obtained in our other surveys using more traditional approaches (in line with previous studies contributing to the literature) demonstrates the feasibility of assessing life-satisfaction using smartphone devices. Future studies might even use this smartphone approach to carry out longitudinal studies with respect to life satisfaction.

Another finding in the present study relates to the association between life satisfaction variables and overall life satisfaction. To obtain a better understanding of the parameters contributing to overall life satisfaction, it seemed appropriate to also analyze the impact of various life satisfaction variables. The variable with the highest impact on overall life satisfaction (Surveys 1–3 in Germany) was leisure. Similar findings were found in previous studies where high satisfaction in leisure had a positive effect on overall satisfaction (12,58). Indeed, there seems to be a link between leisure as a key life satisfaction area and overall life satisfaction. A reason for this finding could be that leisure is becoming more and more important (at least in Western cultures), possibly as a counterpart to daily work or family life (work–life balance is often discussed as an important topic). With the planning and execution of leisure activities, everyone has the chance to design their own private domain and therefore act more autonomously, compared to the areas of work and family where most activities are predetermined (e.g., carrying out work orders and taking care of children necessities). Autonomy has been proposed as one of the core psychological mechanisms [12] enhancing leisure satisfaction and subsequently overall life satisfaction. However, for this to occur, an individual must have the chance to make independent decisions.

With respect to top-down and bottom-up approaches, our current findings suggest that both personality and life satisfaction variables are associated with overall life satisfaction. This view supports an integrative approach towards a model of life satisfaction [13,40], where life satisfaction and personality variables together would contribute to the overall life satisfaction score. It is notable that personality is considered as stable over long periods of time [1], but on the other hand there are various studies showing a close link between personality variables and changes in individual overall life satisfaction [26,59,60]. Therefore, possibly not personality alone but other factors such as various life satisfaction variables are responsible for changes in overall life satisfaction. In this context arguing towards an integrative model of life satisfaction, it is conceivable that the level of life satisfaction is composed of a personality effect representing a stable component over time (trait-like), whereas other factors such as life satisfaction variables, life events, and diseases temporarily modulate the level of life satisfaction (state-like) during the period they are experienced.

After completion of the first two surveys, we continued to focus on the rather moderate influence of personality variables on overall life satisfaction. Although we had some suggestions for this finding (see above), we decided to take a closer look at correlation findings between the BFI-10 and life satisfaction, even though we did not have any evidence to question its reliability. We followed this course of action to insure the absence of a possible bias in our analyses regarding the BFI-10. For this reason, in the third survey, we replaced the BFI-10 [47] with the NEO-FFI [44]. The NEO-FFI was chosen because its psychometric quality has been demonstrated in many studies all around the world. However, the research question of the first two surveys remained the same. Compared to the first two surveys, the results of the stepwise regression analyses did not change with respect to the type of the included variables: life satisfaction variables still explained most of the variance (*R*^2^ = 0.471) compared to demographic and personality variables (*R*^2^ = 0.078). In other words, the personality questionnaire used (BFI-10 vs. NEO-FFI) did not alter our results. This is a favorable result for the psychometric quality of the BFI-10. Furthermore, in Survey 4, where we had our focus on cross-cultural differences in life satisfaction, we administered both the BFI-10 and the NEO-FFI, and the results were quite similar. Therefore, we conclude that the BFI-10 has an adequate psychometric quality as originally suggested by Rammstedt et al. [47]. The advantage of using a shorter questionnaire in terms of the time saved during processing compared to a longer version (and the potential higher number of participants who can therefore be included in a study) is clearly attractive. 

Finally, the fourth survey conducted in China enabled the investigation of possible cross-cultural differences in life satisfaction. As before, the study design and the research question remained the same. Again, in terms of overall life satisfaction, neither personality variables nor demographic variables explained a notable part of the variance. However, in contrast to the findings of the three surveys conducted in Germany, family rather than leisure was the dominate predictor of overall life satisfaction. With respect to the differences between collectivistic cultures, such as in China, and more individualistic cultures, such as in Germany, this result seems understandable. One of the key features of a collectivistic culture is importance of relevant social relationships (e.g., family and friends) and promoting common welfare [25]. Thus, satisfaction with family could therefore have a higher impact on overall life satisfaction than satisfaction with leisure. On the other hand, in a more individualistic culture as Germany, individual and personal interests are of immense importance. From this perspective, the arrangement of and participation in individual leisure activities might be of much greater importance in determining overall life satisfaction, resulting in the different weighting of satisfaction with family and leisure in China and Germany. In this context, we would like to point out that satisfaction with leisure (in addition to other variables) still had a considerable impact on overall life satisfaction in China as well as satisfaction with family in Germany. However, it appears that the impact of life satisfaction variables on overall life satisfaction is different in China and Germany. The result of our fourth survey implies that cross-cultural differences have the potential to influence life satisfaction. Therefore, in future studies, the influence of diverse cultures should be accounted for when investigating or comparing life satisfaction across cultures.

The present study has several strengths but also some limitations. First, we used a cross-sectional design, so it is impossible for us to make inferences about the causality of the relationship between all included variables. Second, we gathered the data by means of electronic devices [61,62] (smartphones, tablets, and personal computers), which excludes any participants not familiar with or not willing to use this kind of technology (however our findings also demonstrate the feasibility of using smartphones for such a research endeavor as the one presented here). Although this could be considered a limitation, it should be kept in mind that the distribution of personal computers, smartphones, and tablets has increased enormously. Moreover, we assume that only people with an affinity for these devices would download an application to use it on their smartphones (Survey 1) or visit an exhibition (Survey 2). Thus the quality of the data, considering the motivation of participants and their ability to use electronic devices, should be excellent. Third, in our second survey, the data were collected within the scope of an exhibition, traveling on a large boat. Since typically only people interested in the topic of the exhibition would consider visiting it, there is a chance that this may have biased our sample. Fourth, all samples differ with respect to their demographic characteristics. The reasons for this are, for example, differences in sample size (ranging from 40,297 to 488 participants), the composition of the samples (students versus non-student samples) and the distribution of gender. Our samples are therefore not fully comparable and the risk of a potential demographic bias exists. Fifth, the life satisfaction variables were ordered following a recommendation of the SOEP to avoid the potential for a biased sample. In doing so the findings could have been possibly influenced by a recency effect (the last item on the list of life satisfaction variables has the highest impact on the judgment of overall life satisfaction). Even though the question concerning overall life satisfaction should be still placed as the final item (SOEP recommendation), it would be advisable (on the basis of our experience from the present study) to counter-balance the life satisfaction domains in future research projects. Finally, for Surveys 1 and 2, we did not gather information on satisfaction with family. This variable was included in Surveys 3 and 4 because it was also one of our goals to investigate cross-cultural effects in terms of life satisfaction. To obtain a more precise view on life satisfaction and for a better comparison of all four surveys, it would have been preferable to have the variable family also included in Surveys 1 and 2.

Besides the limitations mentioned above, there are also several strengths in our study. First, all surveys within this study rely on high sample sizes (up to *N* = 40,297) and for the replications we achieved sample sizes between *N* = 488 and *N* = 4453. Second, the results of the present study are robust because we replicated them in several independent surveys. This is noteworthy, since many research findings do not replicate in psychological science (replication crisis) [63]. Third, the design of the study made it possible to investigate cross-cultural influences in life satisfaction. Furthermore, we were able to investigate the reliability of BFI-10. This goal was achieved in two separate ways: once by replacing the BFI-10 with the NEO-FFI in Survey 3 and secondly by including both questionnaires in Survey 4. This second approach was especially suited for obtaining detailed information concerning the psychometric quality of the BFI-10. Finally, the use of personal computers, smartphones, and tablets to gather information minimizes the occurrence of transcription errors and therefore increases the accuracy of the data.

## 4. Conclusions

In conclusion, taking the data from the present study into account combined with findings in the literature, we propose a life satisfaction model in which overall life satisfaction levels are determined by both top-down and bottom-up effects. The modulations in overall life satisfactions could be caused by bottom-up effects (e.g., life satisfaction variables and life events) but only for the time they are present (note that specific life circumstances have not been assessed in the present work) and not by the influence of personality. This assumption requires further investigation, but evidence from the literature suggests that the presence or absence of specific life circumstances (e.g., disease, healthy lifestyle, and physical activity) and disorders such as pathological buying [64] and Internet addiction [52] have the potential to (temporarily) modify overall life satisfaction. Furthermore, the findings in three of our surveys indicated the prominent role of leisure (at least in Europe) when determining overall life satisfaction. Nevertheless, the dominance of life satisfaction variables on overall life satisfaction might be subject to change in different cultures, as demonstrated in our Chinese sample.

## Figures and Tables

**Table 1:** Mean, standard deviation, minimum, maximum, and skew values of personality (BFI-10) and satisfaction variables for Survey 1; DV = Dependent variable. (*N* = 40,297 except for variable job: *N* = 29,418).

Variables	Mean	SD	Min	Max	Skew
Overall satisfaction (DV)	7.20	2.01	0	10	−0.84
Neuroticism	2.88	0.97	1	5	0.07
Extraversion	3.35	0.94	1	5	−0.21
Openness to Experience	3.46	0.91	1	5	−0.15
Agreeableness	3.06	0.81	1	5	0.04
Conscientiousness	3.17	0.84	1	5	0.09
Health	6.82	2.04	0	10	−0.81
Job	6.53	2.42	0	10	−0.81
Income	5.50	2.79	0	10	−0.41
Housing	6.46	2.18	0	10	−0.49
Leisure	7.21	2.20	0	10	−0.77

**Table 2:** Correlations of personality (BFI-10) and satisfaction variables for Survey 1. (*N* = 40,297 except for variable job: *N* = 29,418).

Variables	N	E	O	A	C	H	J	I	Ho	Le
Overall life satisfaction (OLS)	−0.20 **	0.16 **	0.04 **	0.06 **	0.12 **	0.43 **	0.39 **	0.39 **	0.44 **	0.55 **
Neuroticism (N)		0.21 **	−0.01 **	−0.08 **	−0.11 **	−0.23 **	−0.19 **	−0.13 **	−0.20 **	−0.11 **
Extraversion (E)			0.10 **	0.00	0.10 **	0.14 **	0.13 **	0.06 **	0.18 **	0.13 **
Openness (O)				0.01 **	0.04 **	0.01	0.03 **	0.01	0.03 **	0.03 **
Agreeableness (A)					0.02 **	0.08 **	0.06 **	0.06 **	0.05 **	0.02 **
Conscientiousness (C)						0.12 **	0.20 **	0.14 **	0.06 **	0.09 **
Health (H)							0.34 **	0.26 **	0.36 **	0.31 **
Job (J)								0.50 **	0.30 **	0.28 **
Income (I)									0.24 **	0.26 **
Housing (Ho)										0.39 **

** *p* < 0.001; Leisure (Le).

**Table 3:** Standardized regression coefficient (*β*), *t*-value, and *p*-value for predictors of overall satisfaction score for Survey 1. Personality measured with BFI-10.

Predictors	*β*	*t*	*p*
Leisure	0.360	74.69	<0.001
Income	0.186	36.95	<0.001
Health	0.163	33.33	<0.001
Housing	0.157	31.68	<0.001
Job	0.083	15.99	<0.001
Neuroticism (N)	−0.040	−8.94	<0.001
Extraversion (E)	0.031	6.94	<0.001

**Table 4:** Mean, standard deviation, minimum, maximum, and skew values of personality (BFI-10) and satisfaction variables for Survey 2; DV = Dependent variable. (*N* = 4784 except for variable job: *N* = 4390).

Variables	Mean	SD	Min	Max	Skew
Overall satisfaction (DV)	7.74	2.14	0	10	−1.39
Neuroticism	2.80	0.87	1	5	0.12
Extraversion	3.32	0.95	1	5	−0.16
Openness to Experience	3.58	0.96	1	5	−0.35
Agreeableness	3.09	0.80	1	5	−0.08
Conscientiousness	3.37	0.90	1	5	−0.21
Health	7.30	2.30	0	10	−1.10
Job	7.06	2.47	0	10	−0.95
Income	6.15	3.08	0	10	−0.57
Housing	7.64	2.55	0	10	−1.32
Leisure	7.30	2.35	0	10	−0.97

**Table 5:** Correlations of personality (BFI-10) and satisfaction variables for Survey 2. (*N* = 4784 except for variable job: *N* = 4390).

Variables	N	E	O	A	C	H	J	I	Ho	Le
Overall life satisfaction (OLS)	−0.19 **	0.16 **	0.07 **	0.09 **	0.12 **	0.51 **	0.47 **	0.46 **	0.60 **	0.61 **
Neuroticism (N)		−0.20 **	−0.03	−0.09 **	−0.05 **	−0.21 **	−0.16 **	−0.10 **	−0.13 **	−0.19 **
Extraversion (E)			0.13 **	0.07 **	0.13 **	0.12 **	0.12 **	0.05 **	0.10 **	0.17 **
Openness (O)				0.12 **	0.11 **	0.07 **	0.05 **	0.03 *	0.06 **	0.05 **
Agreeableness (A)					0.09 **	0.11 **	0.08 **	0.07 **	0.11 **	0.09 **
Conscientiousness (C)						0.10 **	0.16 **	0.16 **	0.13**	0.08 **
Health (H)							0.45 **	0.31 **	0.42 **	0.45 **
Job (J)								0.38 **	0.39 **	0.38 **
Income (I)									0.41 **	0.31 **
Housing (Ho)										0.49 **

* *p* < 0.05, ** *p* < 0.001; Leisure (Le).

**Table 6:** Standardized regression coefficient (*β*), *t*-value, and *p*-value for predictors of overall satisfaction score for Survey 2. Personality measured with BFI-10.

Predictors	*β*	*t*	*p*
Leisure	0.322	25.69	<0.001
Housing	0.256	20.29	<0.001
Health	0.148	11.99	<0.001
Income	0.141	12.03	<0.001
Job	0.122	9.79	<0.001
Extraversion	0.040	3.82	<0.001

**Table 7:** Mean, standard deviation, minimum, maximum, and skew values of personality (NEO-FFI) and satisfaction variables for Survey 3; DV = Dependent variable. (*N* = 496).

Variables	Mean	SD	Min	Max	Skew
Overall satisfaction (DV)	7.89	1.84	0	10	−1.49
Neuroticism	2.75	0.65	1.17	4.42	0.32
Extraversion	3.39	0.54	1.42	4.83	−0.36
Openness to Experience	3.56	0.56	2.00	4.83	−0.13
Agreeableness	3.78	0.52	1.67	4.83	−0.65
Conscientiousness	3.80	0.56	1.92	5.00	−0.43
Health	7.59	2.03	0	10	−1.39
Job	3.87	3.90	0	10	0.16
Income	4.19	3.21	0	10	0.07
Housing	7.29	2.01	0	10	−0.99
Leisure	7.03	2.54	0	10	−1.05
Family	7.41	2.49	0	10	−1.20

**Table 8:** Correlations of personality (NEO-FFI) and satisfaction variables for Survey 3. (*N* = 496).

Variables	N	E	O	A	C	H	J	I	Ho	Le	F
Overall life satisfaction (OLS)	−0.23 **	0.23 **	0.02	0.09 *	0.10 *	0.43 **	0.09 *	0.22 **	0.44 **	0.56 **	0.54 **
Neuroticism (N)		−0.48 **	0.04	−0.07	−0.16 **	−0.24 **	−0.08	−0.06	−0.17 *	−0.28 **	−0.12 **
Extraversion (E)			0.02	0.33 **	0.10 *	0.19 **	0.10 *	0.09 *	0.24 **	0.28 **	0.13 **
Openness (O)				0.12 **	−0.12 **	−0.03	0.01	0.06	0.06	0.01	−0.02
Agreeableness (A)					0.10 *	0.02	−0.03	−0.01	0.10 *	0.07	0.08
Conscientiousness (C)						0.04	0.09 *	0.03	0.01	0.06	0.06
Health (H)							0.06	0.08	0.24 **	0.35 **	0.22 **
Job (J)								0.52 **	0.08	0.11 *	0.11 *
Income (I)									0.19 **	0.16 **	0.18 **
Housing (Ho)										0.33 **	0.36 **
Leisure (Le)											0.46 **

* *p* < 0.05, ** *p* < 0.001; Family (F).

**Table 9:** Standardized regression coefficient (*β*), *t*-value, and *p*-value for predictors of overall satisfaction score for Survey 3. Personality measured with NEO-FFI.

Predictors	*β*	*t*	*p*
Leisure	0.285	7.58	<0.001
Family	0.281	7.31	<0.001
Health	0.219	6.33	<0.001
Housing	0.174	4.89	<0.001
Income	0.069	2.09	=0.037

**Table 10:** Mean, standard deviation, minimum, maximum, and skew values of personality NEO-FFI (BFI-10) and satisfaction variables for Survey 4; DV = Dependent variable. (*N* = 488).

Variables	Mean		SD		Min		Max		Skew	
Overall satisfaction (DV)	7.51		2.19		0		10		−0.67	
Neuroticism	2.83	(5.43)	0.69	(1.83)	1.00	(2)	4.67	(10)	0.15	(0.023)
Extraversion	3.30	(6.59)	0.48	(1.63)	1.92	(2)	4.67	(10)	0.14	(0.24)
Openness to Experience	3.37	(7.92)	0.47	(1.76)	2.17	(2)	4.67	(10)	0.12	(−0.61)
Agreeableness	3.62	(7.72)	0.46	(1.60)	2.25	(2)	4.92	(10)	−0.26	(−0.62)
Conscientiousness	3.46	(6.26)	0.54	(1.86)	1.83	(2)	4.92	(10)	0.01	(0.077)
Health	6.93		2.30		0		10		−0.71	
Job	5.55		2.43		0		10		−0.12	
Income	8.12		2.21		0		10		−1.18	
Housing	8.69		1.97		0		10		−1.14	
Leisure	6.96		2.53		0		10		−0.60	
Family	8.16		2.19		0		10		−1.40	

BFI-10 values in brackets.

**Table 11:** Correlations of personality (NEO-FFI) and satisfaction variables for Survey 4. (*N* = 488).

Variables	N	E	O	A	C	H	S	M	Ho	Le	F
Overall life satisfaction (OLS)	−0.23 **	0.19 **	0.14 **	0.17 **	0.19 **	0.31 **	0.36 **	0.44 **	0.45 **	0.45 **	0.55 **
Neuroticism (N)		−0.42 **	-0.04	−0.37 **	−0.41 **	−0.37 **	−0.39 **	−0.20 **	−0.23 **	−0.24 **	−0.30 **
Extraversion (E)			0.06	0.41 **	0.28 **	0.214 *	0.25 **	0.08	0.18 **	0.21 **	0.27 **
Openness (O)				0.05	0.22 **	0.01	0.06	0.08	0.02	0.05	−0.02
Agreeableness (A)					0.30 **	0.16 **	0.20 **	0.12 **	0.18	0.06 **	0.20 **
Conscientiousness (C)						0.31 **	0.35 **	0.17 **	0.11 *	0.19 *	0.23 **
Health (H)							0.53 **	0.19 **	0.18 **	0.34 **	0.32 **
Study (Job) (S)								0.23 **	0.19 **	0.35 **	0.30 **
Money (Income) (M)									0.40 **	0.26 **	0.38 **
Housing (Ho)										0.39 **	0.65 **
Leisure (Le)											0.41 **

* *p* < 0.05. ** *p* < 0.001; Family (F).

**Table 12:** Correlations of personality (BFI-10) and satisfaction variables for Survey 4. (*N* = 488).

Variables	N	E	O	A	C	H	S	M	Ho	Le	F
Overall life satisfaction (OLS)	−0.11 *	0.08 ^1^	0.12 **	0.16 **	0.10 *	0.31 **	0.36 **	0.44 **	0.45 **	0.45 **	0.55 **
Neuroticism (N)		−0.15 **	−0.20 **	−0.22 **	−0.22 **	−0.22 **	−0.24 **	−0.10 *	−0.12 *	−0.16 **	−0.15 **
Extraversion (E)			0.22 **	0.05	0.03	0.03	0.042	0.01	−0.01	0.13 **	0.07
Openness (O)				0.15 **	0.15 **	0.11 *	0.13 **	0.03	0.04	0.06	0.03
Agreeableness (A)					0.14 **	0.16 **	0.18 **	0.09 *	0.21 **	0.09	0.18 **
Conscientiousness (C)						0.25 **	0.27 **	0.12 *	0.09	0.06	0.13 **
Health (H)							0.53 **	0.19 **	0.18 **	0.34 **	0.32 **
Study (Job) (S)								0.23 **	0.19 **	0.35 **	0.30 **
Money (Income) (M)									0.40 **	0.26 **	0.38 **
Housing (Ho)										0.39 **	0.65 **
Leisure (Le)											0.41 **

* *p* < 0.05. ** *p* < 0.001; Family (F). ^1^ On item level the item “… is reserved” showed a null correlation (*r* = 0.02, *p* = 0.617) with overall life satisfaction. Only the item “… is outgoing, sociable” correlated with the overall life satisfaction score (*r* = 0.10, *p* = 0.031).

**Table 13:** Standardized regression coefficient (*β*), *t*-value, and *p*-value for predictors of overall satisfaction score for Survey 4.

Predictors	*β*		*t*		*p*	
Family (Family)	0.363	(0.356)	9.15	(8.96)	<0.001	(<0.001)
Money (Income)	0.216	(0.226)	5.82	(6.06)	<0.001	(<0.001)
Leisure (Leisure)	0.184	(0.187)	4.75	(4.80)	<0.001	(<0.001)
Study (Job)	0.130	(0.125)	3.51	(3.34)	<0.001	(<0.001)
Openness (age)	0.103	(−0.087)	3.01	(−2.55)	=0.003	(=0.011)
Age (Openness)	−0.080	(0.072)	−2.34	(2.10)	=0.020	(=0.036)

Values without brackets: Regression analysis performed with NEO-FFI (values in brackets: Regression analysis performed with BFI-10).

## Supplementary Materials

The following are available online at www.mdpi.com/2076-328X/8/1/1/s1, Table S1: Means and SD of satisfaction variables distinguished by age and gender for survey 1. www.mdpi.com/2076-328X/8/1/1/s2, Table S2: Correlations between NEO-FFI and BFI-10 personality scores for survey 4.

## Appendix A

Chinese version BFI-10（大五调查问卷）

Instruction 指导语：你觉得下述描述是否符合你的性格？

**Table A1:** BFI-10 Chinese Version.

我认为自己是	非常不同意	有点不同意	不确定	有点同意	非常同意
矜持的					
信任别人的					
懒惰的					
放松地、良好地应对压力					
几乎没有艺术的兴趣爱好					
爱与他人交往的					
爱挑剔别人的					
办事周密的					
很容易紧张的					
具有积极的想象力					

English version BFI-10 [47].

Instruction: How well do the following statements describe your personality?

**Table A1:** BFI-10 English Version.

I See Myself as Someone Who …	Disagree Strongly	Disagree a Little	Neither Agree Nor Disagree	Agree a Little	Agree Strongly
... is reserved					
... is generally trusting					
... tends to be lazy					
... is relaxed, handles stress well					
... has few artistic interests					
... is outgoing, sociable					
... tends to find fault with other					
... does a thorough job					
... gets nervous easily					
... has an active imagination					
