# Supplementary material for: Contributing to Overall Life Satisfaction: Personality Traits Versus Life Satisfaction Variables Revisited—Is Replication Impossible?
Source: Behav Sci (Basel). 2017 Dec 23;8(1):1. doi: 10.3390/bs8010001 (PMC5791019; doi:10.3390/bs8010001)
Supplement: Supplementary file 1 [file behavsci-08-00001-s001.pdf]

S1 Table. Means and SD of satisfaction variables distinguished by age for survey 1 (complete sample). (*N* = 40.297)

Variables/age	10 – 19	20 - 29	30 – 39	40 – 49	50 – 59	60 – 69
OLS	7.55 (2.02) (*N* = 16.167)	6.97 (1.97) (*N* = 14.404)	6.82 (1.94) (*N* = 5.813)	7.06 (1.89) (*N* = 2.775)	7.23 (1.99) (*N* = 898)	7.31 (2.10) (*N* = 203)
Health	7.14 (2.04) (*N* = 16.167)	6.74 (1.97) (*N* = 14.404)	6.44 (2.01) (*N* = 5.813)	6.36 (2.10) (*N* = 2.775)	6.30 (2.10) (*N* = 898)	6.35 (2.22) (*N* = 203)
Job	6.50 (2.54) (*N* = 8.616)	6.55 (2.37) (*N* = 11.931)	6.46 (2.38) (*N* = 5.388)	6.66 (2.33) (*N* = 2.570)	6.71 (2.30) (*N* = 787)	6.70 (2.42) (*N* = 116)
Income	5.58 (3.04) (*N* = 16.167)	5.21 (2.65) (*N* = 14.404)	5.68 (2.55) (*N* = 5.813)	5.98 (2.41) (*N* = 2.775)	5.91 (2.53) (*N* = 898)	6.22 (2.43) (*N* = 203)
Leisure	7.65 (2.18) (*N* = 16.167)	6.84 (2.20) (*N* = 14.404)	6.79 (2.11) (*N* = 5.813)	7.29 (2.04) (*N* = 2.775)	7.57 (2.04) (*N* = 898)	7.69 (2.28) (*N* = 203)
Lodging	6.81 (2.21) (*N* = 16.167)	6.35 (2.13) (*N* = 14.404)	5.95 (2.10) (*N* = 5.813)	6.14 (2.07) (*N* = 2.775)	6.21 (2.19) (*N* = 898)	7.00 (2.25) (*N* = 203)

OLS = Overall life satisfaction

S1 Table. Means and SD of satisfaction variables distinguished by age for survey 1 (female). (*N* = 14.874)

Variables/age	10 – 19	20 - 29	30 – 39	40 – 49	50 – 59	60 – 69
OLS	7.22 (2.13) (*N* = 7.464)	6.82 (2.06) (*N* = 5.057)	6.68 (2.08) (*N* = 1.461)	7.18 (2.01) (*N* = 692)	7.58 (1.90) (*N* = 169)	7.17 (2.19) (*N* = 26)
Health	6.78 (2.14) (*N* = 7.464)	6.43 (2.06) (*N* = 5.057)	6.17 (2.16) (*N* = 1.461)	6.17 (2.31) (*N* = 692)	6.43 (2.21) (*N* = 169)	5.94 (2.27) (*N* = 26)
Job	6.29 (2.56) (*N* = 4.018)	6.36 (2.48) (*N* = 4.206)	6.35 (2.52) (*N* = 1.296)	6.83 (2.349 (*N* = 625)	7.01 (2.06) (*N* = 144)	6.50 (2.54) (*N* = 12)
Income	5.29 (3.08) (*N* = 7.464)	4.90 (2.71) (*N* = 5.057)	5.42 (2.71) (*N* = 1.461)	5.86 (2.48) (*N* = 692)	5.83 (2.49) (*N* = 169)	5.88 (2.63) (*N* = 26)
Leisure	7.50 (2.27) (*N* = 7.464)	6.84 (2.32) (*N* = 5.057)	6.72 (2.30) (*N* = 1.461)	7.46 (2.16) (*N* = 692)	7.82 (1.94) (*N* = 169)	8.08 (2.10) (*N* = 26)
Lodging	6.55 (2.26) (*N* = 7.464)	6.15 (2.16) (*N* = 5.057)	5.80 (2.18) (*N* = 1.461)	6.14 (2.16) (*N* = 692)	6.46 (2.15) (*N* = 169)	7.06 (1.99) (*N* = 26)

OLS = Overall life satisfaction

S1 Table. Means and SD of satisfaction variables distinguished by age for survey 1 (male). (*N* = 25.423)

Variables/age	10 – 19	20 - 29	30 – 39	40 – 49	50 – 59	60 – 69
OLS	7.83 (1.87) (*N* = 8.703)	7.05 (1.92) (*N* = 9.347)	6.87 (1.89) (*N* = 4.355)	7.02 (1.84) (*N* = 2.083)	7.15 (2.01) (*N* = 729)	7.33 (2.10) (*N* = 177)
Health	7.44 (1.90) (*N* = 8.703)	6.90 (1.89) (*N* = 9.347)	6.54 (1.95) (*N* = 4.355)	6.42 (2.02) (*N* = 2.083)	6.27 (2.20) (*N* = 729)	6.40 (2.21) (*N* = 177)
Job	6.68 (2.50) (*N* = 4.598)	6.66 (2.31) (*N* = 7.725)	6.50 (2.33) (*N* = 4.092)	6.60 (2.33) (*N* = 1.945)	6.65 (2.35) (*N* = 643)	6.69 (2.42) (*N* = 104)
Income	5.82 (2.97) (*N* = 8.703)	5.38 (2.60) (*N* = 9.347)	5.77 (2.48) (*N* = 4.355)	6.02 (2.39) (*N* = 2.083)	5.92 (2.54) (*N* = 729)	6.27 (2.40 (*N* = 177)
Leisure	7.78 (2.09) (*N* = 8.703)	6.83 (2.13) (*N* = 9.347)	6.81 (2.04) (*N* = 4.355)	7.23 (1.99) (*N* = 2.083)	7.51 (2.06) (*N* = 729)	7.63 (2.30) (*N* = 177)
Lodging	7.03 (2.13) (*N* = 8.703)	6.45 (2.10) (*N* = 9.347)	6.00 (2.08) (*N* = 4.355)	6.14 (2.04) (*N* = 2.083)	6.15 (2.20) (*N* = 729)	7.00 (2.30) (*N* = 177)

OLS = Overall life satisfaction
