# Supplementary material for: Contributing to Overall Life Satisfaction: Personality Traits Versus Life Satisfaction Variables Revisited—Is Replication Impossible?
Source: Behav Sci (Basel). 2017 Dec 23;8(1):1. doi: 10.3390/bs8010001 (PMC5791019; doi:10.3390/bs8010001)
Supplement: Supplementary file 2 [file behavsci-08-00001-s002.pdf]

Table S2. Correlations between NEO-FFI and BFI-10 personality scores.

Variables	EN	ON	AN	CN	NB	EB	OB	AB	CB
NEO-FFI:									
Neuroticism (NN)	-.42***	-0.04	-.37***	-.41***	.62***	-.14**	-.21***	-.26***	-.34***
Extraversion (EN)		.06	.41***	.28***	-.30***	.44***	.24***	.27***	.19***
Openness (ON)			.05	.22***	-.08	.07	.45***	.02	.11*
Agreeableness (AN)				.30***	-.15**	.09	.14**	.45***	.18***
Conscientiousness (CN)					-.30***	.06	.20***	.16***	.67***
BFI-10:									
Neuroticism (NB)						-.15**	-.20***	-.22***	-.22***
Extraversion (EB)							.22***	.05	.03
Openness (OB)								.15**	.15**
Agreeableness (AB)									.14**

* $p < 0.05$; ** $p < 0.01$; *** $p < 0.001$. Note: Different significance level in contrast to all other tables in the article. CB = Conscientiousness BFI-10.
